# Impact of a Formulation Containing Chaga Extract, Coenzyme Q10, and Alpha-Lipoic Acid on Mitochondrial Dysfunction and Oxidative Stress: NMR Metabolomic Insights into Cellular Energy

**DOI:** 10.3390/antiox14060753

**Published:** 2025-06-18

**Authors:** Maria D’Elia, Carmen Marino, Rita Celano, Enza Napolitano, Chiara Colarusso, Rosalinda Sorrentino, Anna Maria D’Ursi, Luca Rastrelli

**Affiliations:** 1 Department of Pharmacy, University of Salerno, Via Giovanni Paolo II, 132, 84084 Fisciano, Italy; mdelia@unisa.it (M.D.); cmarino@unisa.it (C.M.); rcelano@unisa.it (R.C.); enapolitano@unisa.it (E.N.); ccolarusso@unisa.it (C.C.); rsorrentino@unisa.it (R.S.); dursi@unisa.it (A.M.D.); 2 National Biodiversity Future Center—NBFC, 90133 Palermo, Italy; 3 Dipartimento di Scienze della Terra e del Mare, University of Palermo, 90133 Palermo, Italy

**Keywords:** coenzyme Q10, lipoic acid, *Inonotus obliquus*, SH-SY5Y cells, UHPLC-HRMS/MS, ^1^H-NMR metabolomics, mitochondrial dysfunction, cellular metabolism, neurotransmission, fibromyalgia

## Abstract

Objectives: The aim of this study was to evaluate the impact of a novel antioxidant formulation (RE:PAIR, RP-25) containing CoQ10, alpha-lipoic acid, and Chaga extract on mitochondrial dysfunction and oxidative stress. To explore the activity of the formulation on neuronal cells, we explored cell metabolism and its activity as an antioxidant, using a combination of NMR-based metabolomics and UHPLC-HRMS analytical techniques. Methods: SH-SY5Y neuroblastoma cells were treated with RP-25, and cell viability was assessed via CCK-8 assay. Metabolomic profiles of the treated and untreated cells were analyzed by 1D-NMR, providing insights into both intracellular metabolites (endometabolome) and excreted metabolites (exometabolome). Additionally, a UHPLC-HRMS method was developed for quality control and analysis of the RP-25 formulation. Multivariate statistical approaches, including PLS-DA and volcano plot analyses, were used to identify key metabolic changes. Changes in mitochondrial membrane potential were assessed by means of TMRE assay, while radical oxygen species (ROS) were measured by means of the DCHF assay. Results: RP-25 treatment did not affect cell viability but significantly increased metabolic pathways, including amino acid biosynthesis, oxidative phosphorylation, and glycolysis. Higher levels of ATP, glutamate, tyrosine, and proline were observed in treated cells than in control cells, indicating enhanced cellular energy production, as also proved by the increased stability of the mitochondrial membrane after RP-25 treatment, an index of preserved mitochondrial functions. In support, the formulation RP-25 showed antioxidant activity when cells underwent peroxide oxygen stimulation. This effect was mainly due to the combination of Chaga, CoQ10, and ALA, main components of the RP25 formulation. Moreover, the analysis of enriched pathways highlighted that RP formulation influenced mitochondrial energy and oxidative stress response. Conclusions: RP-25 demonstrated biological activity in that it mitigated mitochondrial dysfunction and oxidative stress in neuronal cells, with potential implications in neuronal diseases associated with dysfunctional mitochondria.

## 1. Introduction

Mitochondrial dysfunction has emerged as a central feature of aging and a variety of age-related diseases, including neurodegenerative disorders, cardiovascular diseases, diabetes, cancer, and fibromyalgia [1,2]. Mitochondria, the powerhouses of the cell, are responsible for producing the majority of cellular ATP through oxidative phosphorylation, a process that is highly sensitive to stress and damage. In particular, mitochondrial dysfunction is often associated with inefficient respiration, leading to the overproduction of reactive oxygen species (ROS), which can damage cellular components such as lipids, proteins, and DNA [3,4]. This accumulation of oxidative damage is thought to contribute significantly to cellular senescence, chronic inflammation, and the pathogenesis of numerous diseases.

In recent years, research has increasingly implicated mitochondrial dysfunction in fibromyalgia [5], a chronic pain syndrome that affects millions of people worldwide. Patients with fibromyalgia exhibit altered mitochondrial function, which is believed to contribute to their symptoms, including fatigue, pain, and impaired energy production [6]. Studies have shown that mitochondrial dysfunction in fibromyalgia leads to impaired cellular respiration, exacerbating oxidative stress and inflammation, and contributing to the disease’s hallmark features, including muscle pain and fatigue [7,8]. This has led to growing interest in targeting mitochondrial health as a potential therapeutic strategy for fibromyalgia and other chronic pain disorders.

A promising approach to combat mitochondrial dysfunction and its downstream effects is the use of bioactive compounds with antioxidant and mitochondrial-supportive properties. Among these, coenzyme Q10 (CoQ10), α-lipoic acid, and extracts from medicinal mushrooms have garnered significant attention due to their well-documented beneficial effects on cellular metabolism and mitochondrial health [9]. CoQ10, a vital component of the electron transport chain, has been shown to enhance mitochondrial function and reduce oxidative stress. Similarly, α-lipoic acid, a potent antioxidant, is known to improve mitochondrial efficiency and restore cellular redox balance [10,11]. Chaga mushroom (*Inonotus obliquus*), rich in bioactive compounds such as β-glucans, has also demonstrated promising effects on immune modulation, oxidative stress reduction, and mitochondrial health [12].

In this context, we developed a novel formulation, RP-25, which combines CoQ10, α-lipoic acid, and Chaga extract, along with additional micronutrients such as dihydroquercetin and minerals, designed to enhance mitochondrial function and combat oxidative stress. The aim of this study was to investigate the effects of RP-25 on the metabolism of SH-SY5Y neuroblastoma cells, focusing on its potential to modulate mitochondrial function. Using an untargeted ^1^H-NMR metabolomic approach, we aimed to gain insights into the broader metabolic shifts induced by RP-25 treatment, specifically in relation to mitochondrial respiration and energy production.

Metabolomics, particularly Nuclear Magnetic Resonance (NMR) spectroscopy, has emerged as a powerful tool for studying cellular metabolism and identifying disordered metabolic pathways that underlie various diseases. NMR-based metabolomics provide a non-invasive, comprehensive, and high-throughput method to monitor the entire metabolic profile of cells, revealing subtle changes in cellular metabolism that are often indicative of pathological states [13]. In the context of mitochondrial dysfunction, NMR allows the identification of key metabolic biomarkers and pathways disrupted by oxidative stress, energy dysregulation, or altered cellular function. This makes NMR-based metabolomics an invaluable tool for assessing the metabolic effects of therapeutic interventions and gaining insights into the molecular mechanisms of diseases linked to mitochondrial dysfunction, such as fibromyalgia, neurodegenerative diseases, and cancer.

Through this study, we sought to evaluate the potential of RP-25 as involved in mitigating mitochondrial dysfunction and associated cellular damage, with implications not only for aging and related chronic diseases but also for conditions like fibromyalgia, where mitochondrial dysfunction plays a key role in disease pathophysiology.

## 2. Materials and Methods

### 2.1. Chemical and Standards

Analytical grade ethanol (EtOH), methanol (MeOH), chloroform, acetonitrile (MeCN), tetrahydrofuran (THF), MS grade formic acid (HCOOH), and the reference standards of rutin, hesperidin, quercetin, and coenzyme Q10 (CoQ10) were provided by Merck Chemicals (Milan, Italy). MS grade acetonitrile and water were purchased from Romil (Cambridge, UK). Ultrapure water (18 MΩ) was prepared using a Milli-Q purification system (Millipore, Bedford, TX, USA).

### 2.2. Formulation of RP-25

The formulation of RP-25 consisted of several active ingredients, including Coenzyme Q10 (CoQ10), α-lipoic acid, and Chaga fruit extract (*Inonotus obliquus*) standardized to 25% beta-glucans and dihydroquercetin. These core components were selected for their complementary antioxidant and mitochondrial-supportive properties. The full formulation also contains essential micronutrients (vitamins and trace elements) to support general metabolic function. Specifically, the formulation included CoQ10 at 200 mg, α-lipoic acid at 200 mg, Chaga fruit extract (*Inonotus obliquus*) with 25% beta-glucans at 200 mg, and dihydroquercetin at 50 mg. The Chaga extract used in the formulation was a water-based extract, along with all other components of the RP-25 formulation, kindly supplied by Laboratoarele Medica Srl (Bucharest, Romania). The preparation of RP-25 was carried out at the Department of Pharmacy of the University of Salerno, where all ingredients were measured using an analytical balance for precision. The active components were carefully weighed and combined in a mixing bowl, ensuring thorough mixing to achieve even distribution of the various ingredients. Special care was taken during the blending process to maintain the integrity and efficacy of each component, ensuring a homogenous mixture for optimal formulation. The final product was carefully prepared to meet the required specifications for each ingredient and to guarantee its therapeutic potential.

### 2.3. UHPLC-HRMS/MS Analysis of RP-25 Formulation

The RP-25 formulation was analyzed using a high-resolution UHPLC-HRMS/MS method to profile and characterize its chemical constituents. The samples were dissolved in a hydroalcoholic solution (70% *v*/*v*) with a matrix/solvent ratio of 1:20. Chromatographic analysis was performed using a Vanquish Flex UHPLC system (Thermo Fisher Scientific, Milan, Italy) interfaced with an Orbitrap Exploris 120 mass spectrometer equipped with a heated electrospray ionization source (HESI-II). The system utilized a Kinetex C18 column (100 × 2.1 mm, I.D., 2.6 µm; Phenomex, Bologna, Italy) at a flow rate of 500 µL/min and a column temperature of 30 °C. A 5 µL injection was used for analysis, with the mobile phase consisting of water (A) and MeCN (B), both containing 0.1% formic acid. A gradient elution method was employed, ranging from 2% to 98% MeCN, and each run was followed by a washing and re-equilibration step to ensure system stability. The mass spectrometer was operated in both negative and positive ionization modes, utilizing Full MS/dd-MS2 acquisition for data collection. The resolution was set to 60,000 FWHM for Full MS scans, with fragmentation via stepped HCD (20, 40, and 60 eV) for targeted MS/MS analysis. The detected compounds were identified based on accurate mass, fragmentation patterns, and retention times, with additional confirmation through reference standards or published chemo-taxonomic data. The analysis resulted in the identification of fifteen compounds in the RP-25 formulation, including seven flavonoids, three fatty acids, and four saponins, as detailed in Table 1.

### 2.4. Coenzyme Q10 Analysis

For the determination of CoQ10, a separate UHPLC-UV method was employed. The sample preparation involved extraction with n-hexane (matrix/solvent ratio of 1:20) followed by sonication for 30 min and centrifugation at 13,000 rpm. The supernatant was evaporated under a nitrogen stream, and the residues were reconstituted with the mobile phase (MeCN:THF:Milli-Q water, 50:40:5, *v*/*v*/*v*). The chromatographic separation was achieved using a Kinetex C18 column (100 × 2.1 mm I.D., 2.6 µm; Phenomex, Bologna, Italy) at a flow rate of 400 µL/min, and the analysis was carried out under isocratic conditions with detection at 265 nm. CoQ10 was identified and quantified (procedure reported in Supplementary Materials) by comparing the sample’s UV profile to a reference standard.

### 2.5. Cell Culture

Undifferentiated SH-SY5Y neuroblastoma cells (ATCC, Rockville, MD, USA) were cultured in Dulbecco’s Modified Eagle Medium (DMEM, 4500 mg/mL glucose), supplemented with 10% fetal bovine serum, 2 mM L-glutamine, 100 U/mL penicillin, and 0.1 mg/mL streptomycin. Cells were maintained in a humidified incubator at 37 °C with 5% CO_2_, and they were subcultured every 2 days.

### 2.6. Cell Viability Assay

Cell viability was assessed using the Cell Counting Kit-8 (CCK-8; Dojindo Laboratories, Rockville, MD, USA), which measures the ability of viable cells to reduce the tetrazolium salt WST-8. SH-SY5Y cells were plated in a 96-well plate at a density of 8 × 10^3^ cells/well, allowed to adhere overnight, and then treated with various concentrations of RP-25 (0.80–200 μg/mL) for 24 h. After treatment, CCK-8 solution (10 μL) was added to each well, and absorbance was measured at 450 nm. Cell viability was expressed as the percentage of viable cells relative to untreated controls. Data are presented as mean ± SD from three independent experiments. The statistical significancy was evaluated using the one-way analysis of variance (ANOVA), followed by Bonferroni’s test, with GraphPad Prism 8.0 software (San Diego, CA, USA) assuming significance at *p* < 0.05.

### 2.7. Exposure of Cells to RP-25

SH-SY5Y cells were plated in 100 mm culture dishes (Corning, Corning, NY, USA) and allowed to adhere overnight. The cells were then treated with RP-25 (25 μg/mL) for 24 h. Considering the absence of a significative reduction in cellular viability after the highest RP-25 concentrations administration, we decided to firstly explore the effect of a mild dose of RP-25. The two highest concentrations (70 μg/mL and 200 μg/mL) were excluded since they could induce compensatory metabolic alteration in treated cells, making it hard to understand the effects induced by RP-25 formulation itself. The control group was treated with the vehicle only. At the end of the treatment, the cell medium was collected, and the dishes were washed with cold PBS (pH 7.4) before metabolite extraction.

### 2.8. Measurement of Mitochondrial Membrane Potential

The mitochondrial membrane potential was analyzed by Tetramethylrhodamine, ethyl ester (TMRE; Life Technologies, Monza, Italy) fluorescence. TMRE is cell-permeant, cationic, red-orange fluorescent dye that is readily sequestered by active mitochondria in inverse proportion to the membrane potential [14]. For these experiments, SH-SY5Y were plated (0.4 × 10^5^ cells/well) into MW24-well plates and treated for 4 h with RP25 (25 μg/mL). After the incubation period, TMRE (3.5 nM) was added for 30 min at 37 °C. Cell fluorescence was recorded by means of flow cytometry analysis (BD FacsCalibur, Milan, Italy). Data were expressed as a percentage of TMRE positive cells.

### 2.9. Measurement of Intracellular ROS Production

The formation of intracellular reactive oxygen species (ROS) was evaluated by means of dichlorodihyrofluorescein diacetate (DCHF-DA). DCHF-DA is a cell-permeable probe, which in its initial form is non-fluorescent, whereas, once inside the cell and in contact with ROS, it undergoes multistep conversion forming a fluorescent product, dichlorofluorescein (DCF), detectable by means of flow cytometry [15]. Briefly, SH-SY5Y cells were plated (0.4 × 10^5^ cells/well) into MW24-well plates and treated for 4 h RP-25 (25 μg/mL) or Chaga (8.33 μg/mL), CoQ10 (8.33 μg/mL), α-Lipoic Acid (ALA, 8.33 μg/mL), or their mixture (ratio of each component 1:1:1) ± H_2_O_2_ (1 mM; Sigma-Aldrich, Merck KGaA, Darmstadt, Germany). To note, RP25 or the other treatments were added at 4 h, whereas H_2_O_2_, used as a pro-oxidant stimulus, was added 5 min before the end of treatment [16]. After the subsequent incubation with DCHF-DA 10 μM for 15 min at 37 °C, flow-cytometry analysis was performed (BD FacsCalibur, Milan, Italy). Data were expressed as a percentage of DCHF positive cells.

### 2.10. Sample Extraction

The culture medium was collected and centrifuged at 1000× *g* for 5 min to give information about the metabolites excreted by cells (exometabolome).

The cellular metabolites, which represent the endometabolome, were extracted using a methanol:chloroform:water (1:1:1) mixture. After cell scraping and homogenization, samples were centrifuged at 6000 rpm for 10 min at 4 °C to separate the phases [17]. The polar extracts were dried under vacuum using an SP-Genevac EZ-2 4.0 concentrator, and the resulting extracts were stored at −80 °C prior to NMR analysis.

### 2.11. NMR Spectra Acquisition

Bruker Ascend™ 600 MHz spectrometer was used to acquire the spectra. The spectrometer was equipped with a 5 mm triple resonance Z gradient TXI probe (Bruker Co., Rheinstetten, Germany) at 298 K. TopSpin, version 3.2 was used for the spectrometer control and data processing (Bruker Biospin, Karlsruhe, Germany). All the experiments performed as Nuclear Overhauser Enhancement Spectroscopy (NOESY) 1D were acquired in triplicate. Spectra acquisition was made using 12 ppm spectral width, 20 k data points, presaturation during relaxation delay and mixing time for water suppression [18] and spoil gradient, 5 s relaxation delay, and mixing time of 10 ms. A weighted Fourier transform was applied to the time domain data with a line widening of 0.5 Hz followed by a manual step and baseline correction in preparation for targeted profiling analysis.

### 2.12. Statistical Analysis

NMR spectra of SH-SY-5Ycell cultures eso- and endometabolome were analyzed using an untargeted metabolomic approach. All the spectra were assigned using Chenomx NMR-Suite v8.0 (Chenomx Inc., Edmonton, AB, Canada) and quantified by NMRProcFlow, as previously reported [19]. The quantification matrices reported the metabolites identified and quantified in the eso- and endometabolome of SH-SY-5Y treated and untreated with ST-65 were analyzed using the open-source tool Metaboanalyst 6.0 [20]. The Volcano plot combined T-test and Fold Change performed the univariate approach [21,22]. After normalization by sum log and Pareto, we applied a supervised multivariate approach Partial Least-Squares Discriminant Analysis (PLS-DA) method. The reliability of the supervised model was analyzed using a cross-validation approach, considering the accuracy and parameters Q2 and R2. The metabolites responsible for clusters’ separation in the PLS-DA score plot were classified according to VIP, considering only the metabolites with VIP > 1 [20]. Enrichment Pathways tools were applied to identify the dysregulated biochemical pathways by KEGG database. Only the KEGG paths that reported a rate of false discoveries (FDR) lower than 1, the *p*-value lower than 0.05 and the hits value related to the number of metabolites belonging to the pathway > 1, were chosen [22]. Data are presented as scatter dot plots showing the median. Statistical differences were assessed by means of Mann–Whitney *t* test. *p*-values < 0.05 were considered significant. Statistical analysis was performed by using GraphPad prism 10.4.2 version (San Diego, CA, USA).

## 3. Results

### 3.1. UHPLC-HRMS/MS Analysis

**Figure 1:**
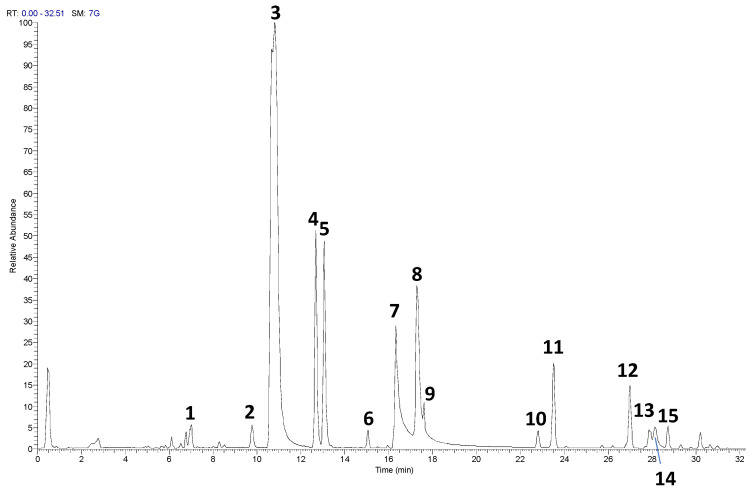
UHPLC−HRMS profile of RP-25 formulation The numbered peaks correspond to the compounds listed in Table 1.

The UHPLC-HRMS/MS analysis of the RP-25 formulation facilitated the identification and characterization of several key bioactive compounds present in the product. Given that the Chaga extract (*Inonotus obliquus*) was incorporated into the formulation, the detected compounds can be attributed not only to the active ingredients added to the formulation (such as CoQ10, α-lipoic acid, and dihydroquercetin) but also to the bioactive constituents derived from the Chaga extract itself. As presented in Table 1 and the corresponding chromatograms (Figure 1), the high-resolution mass spectrometry analysis revealed the presence of several compound classes. Flavonoids: a series of flavonoid derivatives, including quercetin, kaempferol, isorhamnetin, and rutin, were identified in the RP-25 formulation. These compounds are likely derived from the Chaga extract, which is known for its high content of phenolic compounds exhibiting potent antioxidant activity [23]. The MS/MS fragmentation patterns provided clear evidence for the presence of quercetin and kaempferol derivatives, with product ions observed at *m*/*z* 301.0344, 285.0403 (quercetin derivatives) and *m*/*z* 285.0403, 271.0476 (kaempferol derivatives). These findings are consistent with previous reports attributing significant antioxidant activity to these flavonoids in Chaga [24]. The peaks observed in the UHPLC chromatogram at retention times of 9.7, 10.8, 12.6, and 13.0 min correspond to quercetin pentose-rutinoside (**2**), rutin (**3**), kaempferol rutinoside (**4**), and isorhamnetin rutinoside (**5**), as detailed in Table 1. Fatty acids: alpha-lipoic acid (*m*/*z* 205.0362), a key ingredient in the formulation, was readily detected as expected. Additionally, isomers of dodecenedioic acid (compounds **10** and **11**) were identified in the formulation. These compounds are likely derived from the Chaga extract, known to contain various fatty acids and their derivatives [25]. The mass spectra for these compounds (*m*/*z* 227.1289 for dodecenedioic acid isomers) are consistent with those reported in the literature for Chaga-derived fatty acids. The identification of alpha-lipoic acid at retention time 17.3 min and the dodecenedioic acid isomers at 22.8 min and 23.5 min further corroborates the formulation’s composition as outlined in Table 1. Saponins: several saponin-like compounds (**12**–**15**) were detected, with the aglycone moiety identified through characteristic fragmentation patterns. The MS/MS spectra showed a prominent fragment ion at *m*/*z* 439 for compounds **12**, **13**, and **14**, consistent with the presence of a C_30_H_48_O_2_ aglycone, identified as 3-β,22-dihydroxy-lanosta-7,9(11),24-triene. These compounds are likely derived from the Chaga extract, which is known to contain diverse saponins [26]. The observed fragmentation patterns confirm the presence of these compounds. These saponins were eluted between 26.9 min and 28.7 min, as indicated in Table 1. Since the analysis was conducted on the complete RP-25 formulation, the compounds detected, including flavonoids, fatty acids, and saponins, are attributed to the Chaga extract contribution within the formulation. UHPLC-UV analysis was employed to determine the concentration of CoQ10 in the RP-25 formulation. CoQ10 was detected at a concentration of 200 mg per dose, which corresponds to the expected amount in the formulation. The CoQ10 chromatogram (Figure S1) shows a distinct peak at a retention time of 1.3 min, corresponding to CoQ10 [27]. The peak intensity and area under the curve are in full agreement with the expected CoQ10 concentration, thereby confirming that the CoQ10 content in the RP-25 formulation meets the intended specification. The CoQ10 analysis, therefore, validates the accuracy and precision of the formulation preparation, confirming the quality of the final product. The analytical data confirmed that the RP-25 formulation contains the expected active ingredients, CoQ10, α-lipoic acid, and dihydroquercetin, at their specified concentrations. The overall chromatographic and mass spectrometric profile matches with the intended composition of the product, supporting its quality and consistency.

### 3.2. Cell Viability

**Figure 2:**
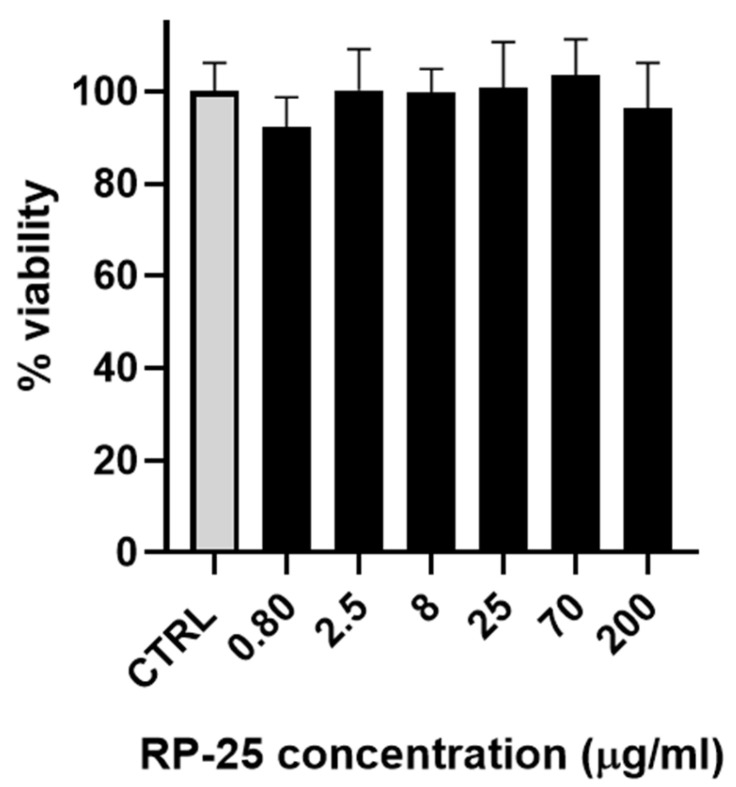
Histogram representing the percentage of viable cells 24 h after treatment with various concentrations of RP-25 (0.80−200 μg/mL). Cell viability was calculated as the percentage of viable cells in treated cultures relative to untreated controls (CTRL). Data are presented as mean ± standard deviation (SD) from three independent experiments. The statistical significance was evaluated using one-way analysis of variance (ANOVA), followed by Bonferroni’s test, with GraphPad Prism 8.0 software (San Diego, CA, USA). Significance was assumed at *p* < 0.05. The statistical test showed the viability of cells exposed to each concentration of RP-25 is not significantly different from the control.

As a preliminary step, the effect of the RP-25 formulation on cell viability was evaluated using SH-SY5Y neuroblastoma cells. Figure 2 illustrates that the RP-25 formulation did not significantly affect the viability of SH-SY5Y cells after 24 h of treatment, even at the highest concentration of 200 μg/mL.

### 3.3. Untargeted ^1^H-NMR Metabolomics Analysis

**Figure 3:**
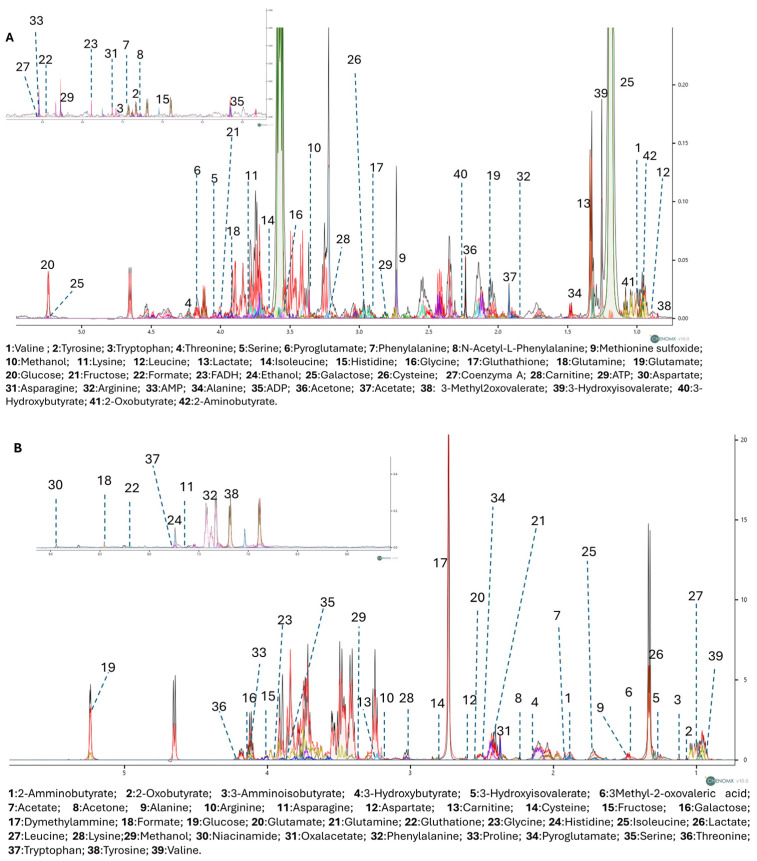
1D-NMR spectra of the endometabolome (**A**) and exometabolome (**B**) of SH-SY5Y cell extracts, showing 42 and 39 metabolites, respectively. Colored lines represent superimposed spectra of different biological replicates from RP-25-treated and control groups.

To further explore the metabolic effects of RP-25, we employed an untargeted ^1^H-NMR metabolomic approach to analyze the endo- and exometabolomes of SH-SY5Y cells, both treated and untreated with RP-25. Using Chenomx NMR-Suite v8.0 (Chenomx Inc., Edmonton, AB, Canada), 42 metabolites were identified in the endometabolome and 39 in the exometabolome (Figure 3A,B). The metabolite quantification was performed relative to the internal standard (TSP).

The quantification matrices were analyzed using MetaboAnalyst 6.0, employing both univariate and multivariate approaches. After normalization (median scaling and log transformation), univariate analysis and multivariate analysis using Partial Least Squares Discriminant Analysis (PLS-DA) were performed [28].

**Figure 4:**
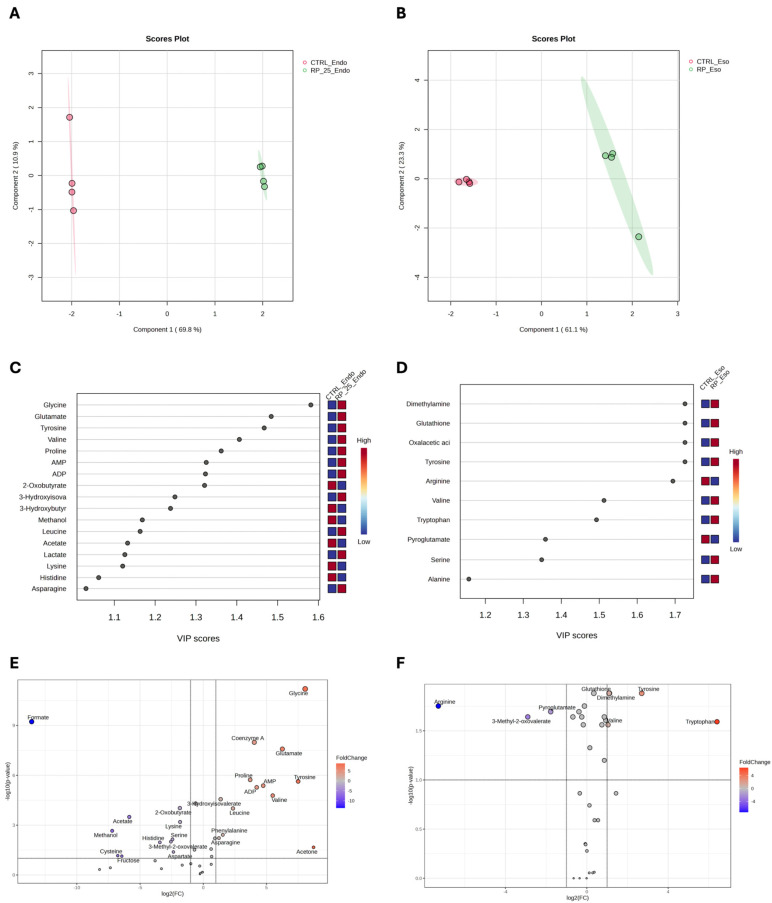
PLS−DA score plots for the endometabolome (**A**) and exometabolome (**B**) of RP-25-treated (green) and untreated (red) cells. VIP scores (**C**,**D**) highlight the metabolites responsible for the clustering observed in both endometabolome and exometabolome. Volcano plots (**E**,**F**) show metabolic changes, with red points indicating upregulated metabolites and blue points indicating downregulated ones. The thresholds of fold change and *p*-value were represented by the dot lines. Only *p*-value < 0.05 and FC ± 2 are considered significant.

Figure 4 presents the results of the PLS-DA analysis, which reveals distinct metabolic changes between RP-25-treated (green) and untreated (red) cells in both the endometabolome (Panel A) and exometabolome (Panel B). The clear separation observed in the PLS-DA score plots suggests significant metabolic differences between the two conditions. Cross-validation analysis confirmed the robustness of the model, with R2 values of 1.0 for both components, indicating perfect model fit, and Q2 values of 0.98 for the endometabolome and 0.86 for the exometabolome, suggesting high predictive power for both datasets [29].

Variable Importance Projection (VIP) analysis (panels C and D) identified key metabolites with VIP scores greater than 1.0, which were responsible for the observed separation between treated and untreated cells. Tyrosine, glycine, glutamate, valine, and proline emerged as major contributors in the endometabolome (panel C), while in the exometabolome, tyrosine, serine, and alanine were identified as the key metabolites driving the clustering (panel D).

The volcano plot confirmed the results of the VIP score analysis and highlighted metabolic changes, with significant upregulation of several metabolites, including glycine, coenzyme A, proline, tyrosine, valine, acetone, AMP, and ATP in the endometabolome of treated cells (Panel E). These alterations reflect a shift in metabolic pathways in response to RP-25 treatment. For example, the increase in tyrosine levels suggests enhanced aromatic amino acid metabolism, while the elevated AMP and ATP levels indicate potential changes in cellular energy status. Additionally, the decrease in formate levels in treated cells may indicate altered one-carbon metabolism. In the exometabolome (Panel F), tyrosine, tryptophan, and serine were significantly upregulated, while arginine levels were decreased in the medium of treated cells. These changes suggest modifications in amino acid metabolism that could be associated with the cellular response to RP-25.

### 3.4. Enrichment Pathway Analysis

Enrichment pathway analysis confirmed the impact of RP-25 on several metabolic pathways, particularly those related to neurotransmission and cellular energy metabolism. Notably, dysregulated pathways included tyrosine metabolism, phenylalanine metabolism, and phenylalanine–tyrosine–tryptophan biosynthesis, as well as glutamate, alanine, aspartate, and glycolysis/gluconeogenesis metabolism (Table 2). Notably, RP-25 treatment led to a significant increase in ATP concentrations, as demonstrated by NMR analysis, suggesting enhanced cellular energy production and potential effects on oxidative phosphorylation and cellular energy regulation.

### 3.5. ATP Concentration and Mitochondrial Membrane Potential Analysis

**Figure 5:**
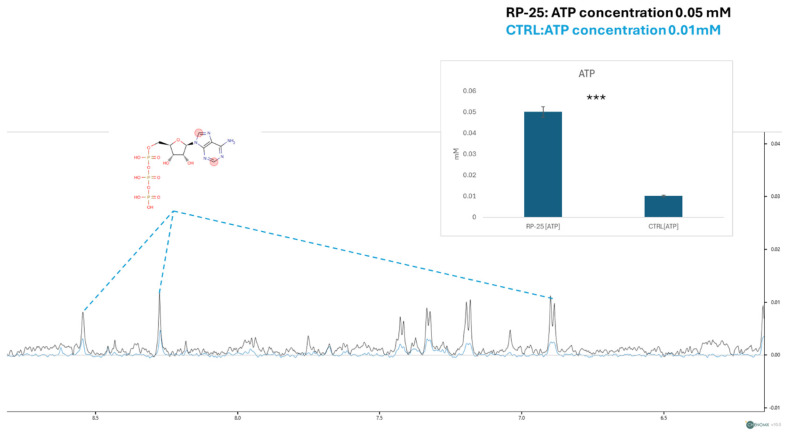
ATP concentration analysis in RP-25-treated SH-SY5Y cells. NMR spectra showing ATP signal intensities in the endometabolome of treated and untreated cells. The quantified ATP concentrations (mean ± SEM) are based on TSP-d4 normalization. The prontons responsible for the NMR-spectra signals are labelled in the red cycles of ATP structure *** denote respectively *p* < 0.001 vs. CTRL.

In addition to the overall metabolic profile changes, we focused on the concentration of ATP, a key molecule involved in cellular energy metabolism. The figure below shows a zoom-in on the spectral window representing the ATP signals. Using the TSP signal as an internal standard for quantification, we observed a decrease in ATP signal intensities in the endometabolome spectra of control cells (in blue) and an increase in the spectra of cells treated with the RP-25 extract. The ATP concentration was then quantified using Chenomix software in all samples, yielding an average concentration of 0.05 mM in the treated cell extract and 0.01 mM in the untreated cell extract. The ATP concentration is presented as the mean mM concentration value ± SEM from two independent experiments, with the average calculated from three replicates, based on TSP-d4 peak normalization. The resulting *p*-value of 7.52 × 10^−3^ indicates a significant increase in ATP levels in RP-25-treated cells (Figure 5).

**Figure 6:**
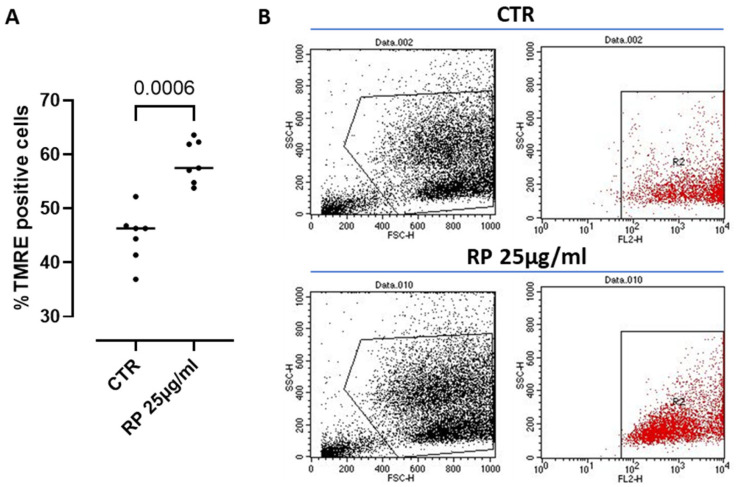
Mitochondrial membrane potential analysis in RP-25-treated SH-SY5Y cells. (**A**) Cells were treated for 4 h with RP-25 (25 µg/mL), and flow cytometry analysis was carried out through tetramethylrhodamine ethyl ester (TMRE) assay. Scatter dot dots represent the percentage of TMRE positive; data are expressed as median (**A**). Representative dot plots show, on the left, the forward and side scatter analysis (FSC and SSC) of SH-SY5Y cells, and. on right, cells labeled with TMRE (**B**). Statistical differences were assessed by means of Mann–Whitney *t* test.

The mitochondrion is known to be the major ATP-producing organelle of the cell via the oxidative phosphorylation (OXPHOS) [30]. Therefore, in order to monitor ATP production and its correlation with the mitochondrial function under RP-25 treatment, we went on by analyzing possible changes in the mitochondrial membrane depolarization, a well-known parameter to assess key mitochondrial processes and cell homeostasis [31]. To this aim, we used the cationic dye TMRE, which accumulates in active mitochondria due to their relative negative charge leading to an increased membrane potential; instead, inactive mitochondria do not retain TMRE and show a decreased membrane potential. We found that treatment with RP-25 for 4 h significantly increased the percentage of TMRE positive cells compared to control (CTR) cells (Figure 6), suggesting the ability of RP-25 to regulate and stabilize the mitochondrial membrane potential. Treatment of cells with RP-25 was also evaluated at 8 h. In particular, higher TMRE positive cells were observed after RP-25 treatment, although it did not reach statistical differences (ctr: 52.78 ± 2.012 vs. RP-25: 61.35 ± 1.8).

**Figure 7:**
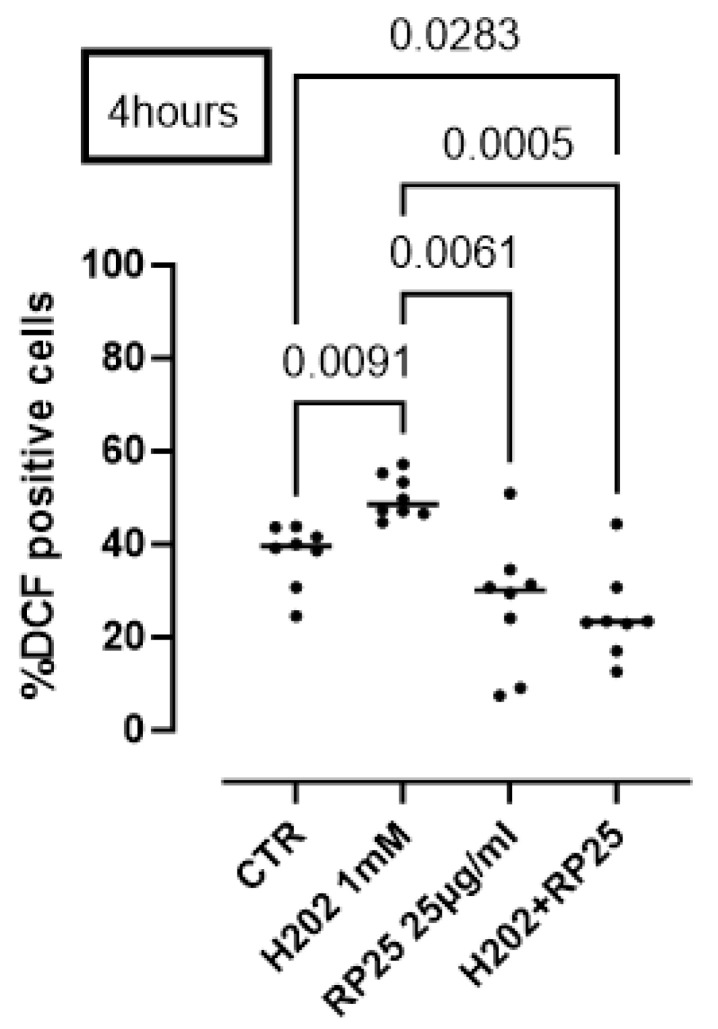
Antioxidant activity of RP25 formulation. Cells were treated for 4 h with RP-25 (25 µg/mL). H_2_O_2_ was added as a pro-oxidant stimulus. DCHF assay was used and flow cytometry analysis was carried out. Statistical differences were assessed by means of one-way ANOVA followed by Tukey’s multiple comparison post test.

To further prove the antioxidant activity of RP25 formulation, we performed a DCHF assay. Cells were induced to oxidative stress using H_2_O_2_ (1 mM). The pretreatment of cells with RP25 significantly avoided ROS production by H_2_O_2_ (Figure 7).

**Figure 8:**
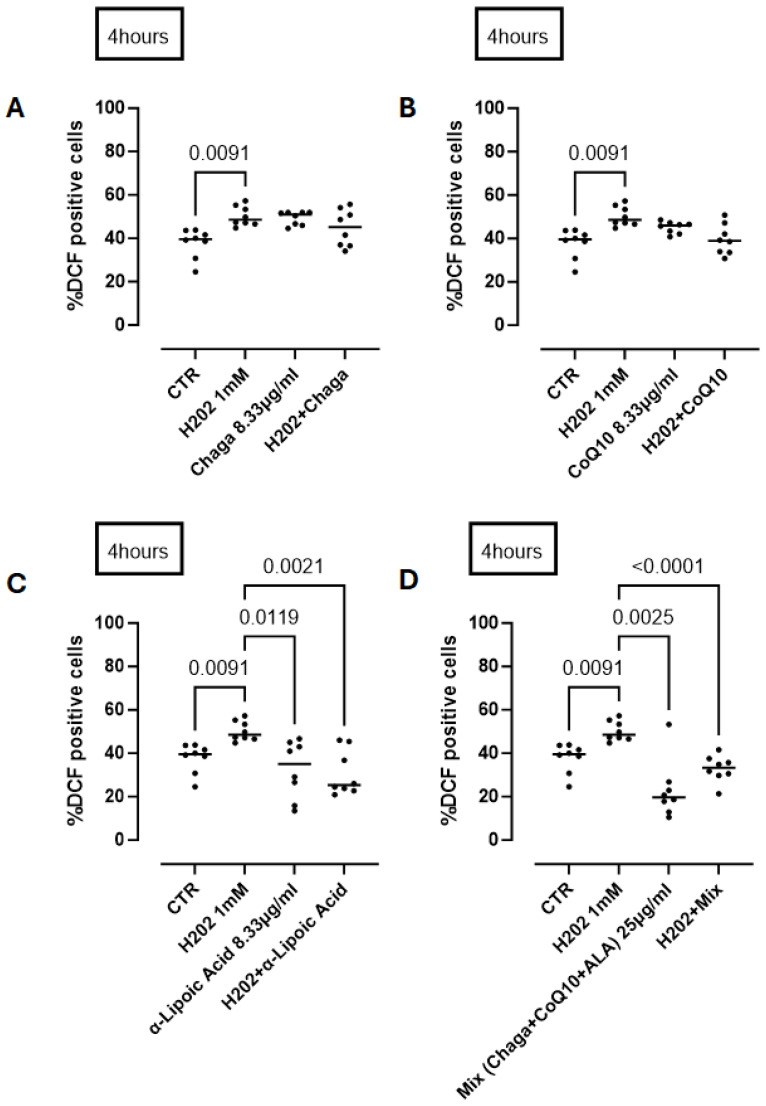
Antioxidant activity of the single components of RP25 formulation and their mixture (mix) at a ratio of 1:1:1. Cells were treated for 4 h with Chaga (**A**), CoQ10 (**B**), α-lipoic acid (**C**) and their mixture (**D**). H_2_O_2_ was added as a pro-oxidant stimulus. DCHF assay was used and flow cytometry analysis was carried out. Statistical differences were assessed by means of one-way ANOVA followed by Tukey’s multiple comparison post test.

In addition to understanding the activity of its single component of RP25 formulation we treated cells with Chaga (8.33 µg/mL) or CoQ10 or ALA (α-lipoic acid) in the same manner as for RP-25 treatment. We found that Chaga (Figure 8A) and CoQ10 (Figure 8B) alone did not reduce ROS production at 4 h after H_2_O_2_ treatment. In contrast, ALA was able to significantly reduce ROS production by neural cells (Figure 8C). The addition of the three components as in RP25, thus respecting the same component ratio, still had a significant antioxidant effect (Figure 8D), similar to that observed by RP25 (Figure 7) (RP25: 24.75 ± 3.37 vs. 32.89 ± 2.15% DCF positive cells).

## 4. Discussion

This study aimed to evaluate the effects of the RP-25 formulation, composed of CoQ10, α-lipoic acid, Chaga extract, and micronutrients, on the mitochondrial function and metabolic profiles of SH-SY5Y neuroblastoma cells, using untargeted ^1^H-NMR metabolomics. The results demonstrate that RP-25 treatment induces significant changes in cellular metabolism, providing valuable insights into its potential therapeutic effects, particularly in the context of mitochondrial dysfunction and diseases associated with oxidative stress, including neurodegenerative disorders and fibromyalgia.

### 4.1. RP-25 Formulation

The RP-25 formulation is a complex blend of active ingredients, including CoQ10, alpha-lipoic acid, dihydroquercetin, Chaga extract, and essential micronutrients. Each component contributes to the overall cellular bioenergetic and antioxidant profile of the product. Analytical characterization using UHPLC-HRMS/MS and HPLC-UV confirmed the presence of all targeted compounds and revealed additional bioactive constituents derived from the Chaga extract, highlighting the formulation’s complexity. The synergistic interaction between these components aims to improve mitochondrial efficiency, reduce oxidative stress, and support cellular integrity across multiple biological systems.

Coenzyme Q10 (CoQ10) is a crucial component of the mitochondrial electron transport chain, functioning within complex I and III as an electron carrier. It facilitates the transfer of electrons from NADH and FADH2 to oxygen, driving ATP production via oxidative phosphorylation. Beyond its role in bioenergetics, CoQ10 is a potent lipophilic antioxidant, stabilizing cellular membranes and scavenging reactive oxygen species (ROS), thereby mitigating oxidative damage at the cellular level [32]. The decline in CoQ10 levels with age is associated with mitochondrial dysfunction and increased oxidative stress, contributing to cellular senescence and age-related diseases [33]. RP-25 includes CoQ10 at 200 mg per dose, as confirmed by UHPLC-UV analysis, ensuring sufficient concentrations to support mitochondrial bioenergetics and antioxidant defenses [34,35].

Alpha-lipoic acid (ALA), a sulfur-containing fatty acid, serves both as a cofactor for mitochondrial enzymes and as a multifaceted antioxidant. ALA supports mitochondrial bioenergetics by acting as a coenzyme for pyruvate dehydrogenase, an enzyme complex crucial for converting pyruvate into acetyl-CoA, thus facilitating the entry of carbon substrates into the citric acid cycle. This optimizes NADH and FADH2 production, and these feed into the electron transport chain for ATP synthesis. ALA also regenerates other antioxidants, including vitamins C and E, by reducing their oxidized forms [36,37]. This regenerative effect amplifies cellular antioxidant capacity, particularly in high-oxidative stress environments. In RP-25, ALA is included at 200 mg per dose, which has been shown to restore mitochondrial function, enhance cellular redox balance, and reduce inflammatory markers associated with aging [38]. The UHPLC-HRMS analysis confirmed its incorporation at the expected retention time of 17.3 min, ensuring its optimal concentration for cellular protection and energy metabolism.

A key feature of RP-25 is the inclusion of Chaga extract (*Inonotus obliquus*), which contains a range of bioactive compounds, including flavonoids, polyphenols, polysaccharides, and terpenoids, each contributing to the formulation’s antioxidant, anti-inflammatory, and immunomodulatory effects [39,40]. Flavonoid derivatives such as quercetin, kaempferol, and isorhamnetin, identified through UHPLC-HRMS/MS, are potent antioxidants that scavenge free radicals and modulate intracellular signaling pathways regulating oxidative stress. These flavonoids inhibit ROS production and enhance the activity of antioxidant enzymes like superoxide dismutase (SOD) and catalase, thus reducing oxidative damage [41,42]. Chaga’s polysaccharides activate the Nrf2-Keap1 pathway, which orchestrates the transcriptional activation of phase II detoxifying enzymes like quinone reductase 1 (NQO1) and glutathione S-transferase (GST). This upregulation enhances the cell’s antioxidant capacity, facilitating ROS neutralization and detoxification, thus maintaining cellular redox homeostasis and mitigating oxidative stress [43]. These mechanisms suggest RP-25’s potential in protecting against oxidative damage, supporting cellular longevity, and reducing the risk of chronic degenerative diseases by enhancing cellular defense systems and maintaining redox balance. Additionally, Chaga extract contributes bioactive fatty acids, such as dodecenedioic acid, which help regulate cellular membrane fluidity and stability. These fatty acids maintain membrane integrity and enhance membrane-bound enzyme functions, essential for proper cell signaling and metabolic processes [44]. The identification of saponins (compounds **12**–**15**) in RP-25 further supports its antioxidant and immune-modulating properties. Saponins interact with cellular membranes and modulate immune responses by influencing macrophage and dendritic cell activity. These compounds inhibit the activation of nuclear factor-kappa B (NF-κB), a transcription factor regulating pro-inflammatory cytokine expression, thus reducing systemic inflammation and promoting tissue homeostasis [45]. Finally, dihydroquercetin (taxifolin), a flavonoid with vascular protective and antioxidant properties, enhances microcirculation and collagen synthesis. It modulates vascular markers like vascular endothelial growth factor (VEGF), improving capillary function and nutrient delivery to tissues. This action also improves collagen cross-linking and maintains tissue elasticity, crucial for cellular function and preventing age-related tissue degeneration [46,47].

### 4.2. Impact on Mitochondrial Function and Energy Metabolism

A key finding of this study is the modulation of cellular energy metabolism following RP-25 treatment, as revealed by ^1^H-NMR metabolomics. Notably, RP-25 treatment resulted in a significant increase in intracellular ATP concentrations, with a measured average concentration of 0.05 mM in treated cells compared to 0.01 mM in untreated cells, indicating enhanced cellular energy production [48]. These changes suggest a potential improvement in mitochondrial bioenergetics, with RP-25 likely promoting oxidative phosphorylation and ATP synthesis. The increased ATP levels, in particular, suggest a more efficient mitochondrial energy production, which is critical for maintaining cellular function. This observation is consistent with the well-known roles of CoQ10 and α-lipoic acid in enhancing mitochondrial efficiency.

The observed increase in ATP concentrations in RP-25-treated cells suggests a positive impact of the formulation on cellular bioenergetics, potentially enhancing mitochondrial function and supporting energy metabolism in the context of cellular stress. Mitochondria are complex cellular structures responsible for producing ATP and play a key role in several biological processes, including calcium homeostasis, thermogenesis, apoptosis [49], ROS signaling and redox modulation [50]. As previously reported, mitochondrial membrane polarization is a parameter to assess key mitochondrial function and cell homeostasis [28]. Herein, it is well known the direct correlation between mitochondrial membrane potential and the OXPHOS-fueled ATP production. RP-25 increased ATP levels at 24 h when added to neuronal cells. In support, RP-25 was able to stabilize the mitochondrial membrane potential (Figure 6). The effect of this activity was further supported by RP25 antioxidant activity when cells were treated with a pro-oxidant stimulus (Figure 7). In addition, the metabolomic analysis revealed significant shifts in key metabolic pathways involved in energy metabolism, particularly glycolysis, gluconeogenesis, and pyruvate metabolism. Specifically, RP-25 treatment was associated with a notable increase in oxaloacetate excretion and a reduction in ketone body levels, including 3-hydroxybutyrate, acetate, and 2-oxobutyrate. These metabolic changes suggest a reprogramming of cellular metabolism towards more efficient ATP production, potentially via enhanced glycolytic activity and reduced reliance on fatty acid oxidation pathways, including ketogenesis. The observed shift from ketogenesis to glycolysis is indicative of a more efficient energy production mechanism, possibly facilitated by RP-25’s ability to optimize mitochondrial function/membrane potential and antioxidant activity [51].

The metabolic changes observed were further validated by enrichment pathway analysis, which identified significant dysregulation in energy-producing pathways, such as glycolysis, gluconeogenesis, and pyruvate metabolism. These alterations in key metabolic routes suggest that RP-25 may help restore metabolic homeostasis in cells affected by mitochondrial dysfunction, a hallmark of diseases like fibromyalgia. In fibromyalgia, mitochondrial inefficiency and disrupted cellular energy production contribute significantly to the pathophysiology of the disease, manifesting as fatigue, pain, and cognitive dysfunction [8,52,53]. The observed modulation of energy pathways in response to RP-25 treatment offers a promising avenue for therapeutic intervention in such conditions. Moreover, the increase in ATP production following RP-25 treatment raises an intriguing possibility related to cellular differentiation processes. Previous studies have demonstrated that differentiation of SH-SY5Y cells is associated with increased mitochondrial respiration and ATP production [54,55,56]. This possibility warrants further investigation to determine whether the RP-25 formulation could promote differentiation in SH-SY5Y cells, which may enhance cellular bioenergetics and mitochondrial function.

Taken together, these metabolomic findings support the hypothesis that RP-25 may optimize cellular metabolism by improving mitochondrial function and energy production. These changes hold significant therapeutic potential for conditions characterized by mitochondrial dysfunction and impaired energy metabolism, such as fibromyalgia. Further studies are needed to confirm these effects and elucidate the specific molecular mechanisms by which RP-25 influences mitochondrial activity and overall metabolic regulation, including the potential impact on cellular differentiation pathways. Taken together, these metabolomic findings support the hypothesis that RP-25 may optimize cellular metabolism by improving mitochondrial function and energy production. These changes hold open new perspectives in diseases where mitochondrial dysfunction/oxidative stress and impaired energy metabolism are crucial. Further studies are needed to confirm these effects and elucidate the specific molecular mechanisms by which RP-25 influences mitochondrial activity and overall metabolic regulation.

### 4.3. Amino Acid Metabolism

Our NMR analysis revealed significant changes in amino acid metabolism, particularly in neurotransmitter synthesis. RP-25 treatment led to the upregulation of amino acids such as tyrosine, glutamate, glycine, proline, and valine, which are essential for neurotransmitters like dopamine, norepinephrine, and serotonin, all critical for brain function [57]. Tyrosine and phenylalanine metabolism were notably affected, as shown by the Variable Importance in Projection (VIP) analysis. The increase in tyrosine and its metabolites suggest that RP-25 may enhance dopamine and norepinephrine biosynthesis, both involved in mood regulation and cognitive function. Tyrosine is also a precursor for thyroid hormones, important for metabolic regulation and neuroprotection [58]. Elevated levels of glutamate and glycine, key excitatory and inhibitory neurotransmitters, respectively, suggest that RP-25 may enhance synaptic plasticity and neuronal activity. Glutamate is crucial for synaptic transmission, learning, and memory, while glycine modulates excitatory neurotransmission [59]. These changes indicate improved neurotransmission and synaptic function, which are vital for cognitive and motor function, especially in neurodegenerative conditions. Changes in the exometabolome, such as increases in alanine, serine, and tryptophan, further suggest that RP-25 influences brain metabolism. Alanine and serine are involved in protein and neurotransmitter synthesis, while tryptophan is a serotonin precursor, regulating mood, sleep, and appetite [60]. These findings suggest that RP-25 not only affects neurotransmitter synthesis but also broader brain-related metabolic pathways. The results are consistent with previous studies on the neuroprotective effects of α-lipoic acid and CoQ10. Both compounds have shown benefits in supporting brain health through their antioxidant properties and by improving mitochondrial function in neurons [61]. The upregulation of neurotransmitter-related metabolites like glutamate, glycine, and tyrosine further supports the idea that RP-25 could help restore neurochemical balance and neuronal communication, making it a promising candidate for treating conditions with neurotransmission dysregulation, such as Alzheimer’s, Parkinson’s, and fibromyalgia [62,63]. Given the observed increase in intracellular ATP and the upregulation of amino acids involved in neurotransmitter biosynthesis (e.g., tyrosine, glutamate, glycine), it is plausible that RP-25 may influence pathways related to neuronal differentiation. While this hypothesis was not directly tested in the present study, future investigations employing specific differentiation markers and morphological analyses will be essential to explore this potential neurotrophic effect.

### 4.4. Oxidative Stress and Redox Balance

RP-25 treatment led to significant changes in metabolites related to cellular redox regulation, as revealed by our metabolomic analysis. Notably, RP-25 resulted in a reduction in formate levels and an increase in coenzyme A and proline, which are involved in redox homeostasis [13,64,65]. Formate, which is linked to mitochondrial dysfunction and oxidative stress, decreased in RP-25-treated cells, suggesting improved mitochondrial function and redox balance. While ROS levels were not directly measured, these metabolic changes, combined with the known antioxidant properties of α-lipoic acid and CoQ10, suggest that RP-25 may reduce oxidative damage [66]. The increase in coenzyme A levels indicates enhanced mitochondrial function. Coenzyme A plays a vital role in the Krebs cycle, which is crucial for ATP production and ROS detoxification [67]. This is consistent with previous studies showing that α-lipoic acid and CoQ10 help support mitochondrial bioenergetics and reduce oxidative stress by aiding electron transport in the mitochondrial respiratory chain [47]. Additionally, the increase in proline levels suggests that RP-25 may enhance cellular antioxidant capacity. Proline plays a role in collagen synthesis and redox regulation, as it acts as an antioxidant by scavenging ROS [68]. The higher proline levels observed here imply that RP-25 may increase the activity of antioxidant enzymes and aid in the repair of oxidative damage. Pathway enrichment analysis also revealed involvement in glutathione metabolism, a key antioxidant that neutralizes ROS and maintains redox homeostasis [69]. Glutathione dysregulation is common in diseases like fibromyalgia, where oxidative stress contributes to chronic pain and fatigue [8,70]. Our findings suggest that RP-25 may help restore glutathione balance, improving the cell’s ability to manage oxidative stress. The observed changes in formate, coenzyme A, and proline levels support the idea that RP-25 may improve redox balance and mitochondrial efficiency.

To further validate these findings under conditions of oxidative challenge, we conducted additional experiments in which SH-SY5Y cells were exposed to hydrogen peroxide (H_2_O_2_) as a pro-oxidant stimulus. The results, presented in Figure 7 and Figure 8, confirmed that RP-25 significantly attenuated ROS accumulation, and that this effect was more pronounced than that of its individual components, supporting the synergistic antioxidant activity of the full formulation.

Beyond the above metabolomic data, our in vitro results suggested that RP-25 is associated with an improvement of the mitochondrial functions as demonstrated by the maintenance of mitochondrial membrane potential and the ensuing increase in ATP production. Thus, according to the involvement of the mitochondrion in redox modulation and ROS signaling, we showed that RP-25 should be able to protect from oxidative damage in that it enhances the efficiency of mitochondrial energy metabolism. In support of this concept, a component of RP-25 formulation, CoQ10, has been proved to stabilize the mitochondrial inner membrane structure, protecting the mitochondria from oxidative damage [71], thus exhibiting antioxidant properties by neutralizes free radicals, reducing ROS generation and inhibiting oxidative stress-induced damage [72].

### 4.5. Potential Therapeutic Implications for Mitochondrial Diseases and Fibromyalgia

Our study suggests that RP-25 shows potential benefit for improving mitochondrial function, restoring metabolic balance, and reducing oxidative stress in cells with mitochondrial dysfunction. Given the critical role of mitochondrial health in diseases like neurodegenerative disorders, cardiovascular issues, and fibromyalgia, RP-25 may provide a novel treatment/adjuvant approach. Clinical trials [8,53] have highlighted the role of mitochondrial dysfunction in fibromyalgia, revealing alterations in amino acid metabolism and energy production pathways. Specifically, we observed changes such as decreased phenylalanine and increased isoleucine, disrupting neurotransmitter synthesis [53]. Additionally, we identified impaired ATP production and dysregulation of metabolites like proline and formate, which are crucial for mitochondrial function. These findings indicate that mitochondrial dysfunction in fibromyalgia is closely tied to metabolic disruption, contributing to symptoms like fatigue and pain. RP-25, through its combination of CoQ10, α-lipoic acid, Chaga, and other components, may improve mitochondrial bioenergetics by stabilizing the mitochondrial membrane potential and exerting antioxidant activities, enhancing electron transport and restoring redox balance, promoting more efficient oxidative phosphorylation. This could address the mitochondrial dysfunction in fibromyalgia, as demonstrated in our trials, where metabolic interventions alleviated symptoms like fatigue and muscle pain [8,53]. RP-25’s effect on amino acids, such as glutamate and proline, may also help restore balance in neurotransmission and cellular metabolism, further alleviating neurological and muscular symptoms.

## 5. Conclusions

Our study provides strong evidence that RP-25 significantly impacts cellular metabolism, particularly mitochondrial function, energy production, and, potentially, oxidative stress regulation. By modulating key metabolic pathways involved in energy metabolism, neurotransmission, and redox balance, RP-25 emerges as a promising adjuvant for diseases associated with mitochondrial dysfunction, such as fibromyalgia. The use of ^1^H-NMR-based metabolomics has provided valuable insights into the complex metabolic changes induced by RP-25, revealing its potential to identify biomarkers and therapeutic targets. A notable strength of this study is the comprehensive application of advanced metabolomic techniques, which allowed us to capture a wide range of metabolic alterations and gain important mechanistic insights. These findings contribute to the growing understanding of mitochondrial dysfunction in chronic diseases and highlight RP-25’s potential to address these disturbances. However, some limitations should be considered. Although our in vitro results are encouraging, clinical studies are required to confirm the therapeutic effects of RP-25 in human populations. Additionally, while ^1^H-NMR profiling provided valuable information on metabolic changes, incorporating other omics approaches in future research may offer a more comprehensive understanding of its molecular impact. Furthermore, further work is needed to confirm RP-25’s direct impact on mitochondrial function and oxidative stress, particularly under conditions of induced oxidative damage. RP-25 appears to modulate energy metabolism, potentially improving cellular bioenergetics and neurotransmitter synthesis, but additional studies using oxidative stress inducers and direct mitochondrial assays are necessary to fully assess its therapeutic potential. In conclusion, RP-25 demonstrates significant promise as an adjuvant agent for conditions related to mitochondrial dysfunction, including fibromyalgia. Further clinical trials are necessary to validate these findings and fully explore RP-25’s therapeutic potential.

## Figures and Tables

**Table 1:** UHPLC-HRMS data of compounds detected in the RP-25 formulation.

N. ^a^	RT [min].	*m*/*z*	Formula	ppm	MS/MS	Name
1	7.0	443.0696	C_32_H_11_O_3_	1.424	221, 125, 80	unknown
2	9.7	741.188	C_32_H_37_O_20_	1.053	300/301	Quercetin pentose-rutinoside
3	10.8	609.1457	C_27_H_29_O_16_	1.213	300/301	Rutin ^b^
4	12.6	593.1509	C_27_H_29_O_15_	1.422	284/285, 255, 227	Kaempferol rutinoside
5	13.0	623.1613	C_28_H_31_O_16_	1.057	315, 299, 271	Isorhamnetin rutinoside
6	15.0	253.0506	C_15_H_9_O_4_	4.563		Dadzein ^b^
7	16.3	301.0353	C_15_H_9_O_7_	3.425	179, 151	Quercetin ^b^
8	17.3	205.0362	C_8_H_13_O_2_S_2_	5.084	171, 127	lipoic acid
9	17.6	269.0456	C_15_H_9_O_5_	4.349		Apigenin ^b^
10	22.8	227.1289	C_12_H_19_O_4_	5.127	209, 183, 165	Dodecenedioic acid isomer
11	23.5	227.1288	C_12_H_19_O_4_	4.686	209, 183,165	Dodecenedioic acid isomer
12	26.9	941.5097	C_48_H_77_O_18_	0.713	439	lanostane-type saponin
13	27.8	795.4530	C_42_H_67_O_14_	0.587	439	lanostane-type saponin
14	27.9	911.5001	C_47_H_75_O_17_	0.332	439, 421	lanostane-type saponin
15	28.7	925.5159	C_48_H_77_O_17_	0.435	423	lanostane-type saponin

^a^ Compounds are numbered according to their elution order. ^b^ Compared with reference standards.

**Table 2:** Enrichment pathway analysis of significant biochemical pathways affected by RP-25 treatment. Pathways with more than two metabolites and *p*-value and False Discovery Rate (FDR) values below 0.05 are considered significant.

	Hits	*p*-Value	FDR
Pantothenate and CoA biosynthesis	4	9.56 × 10^−5^	3.82 × 10^−3^
Glyoxylate and dicarboxylate metabolism	6	4.60 × 10^−2^	9.20 × 10^−1^
Tyrosine metabolism	3	4.63 × 10^−1^	0.00046293
Lipoic acid metabolism	3	0.00052912	0.0035275
Histidine metabolism	3	0.0015907	0.0079534
Glutathione metabolism	5	0.0018923	0.0084101
Phenylalanine metabolism	2	0.0028912	0.0096374
Phenylalanine tyrosine and tryptophan biosynthesis	2	0.0028912	0.0096374
Glycolysis/Gluconeogenesis	2	0.0060371	0.017249
Pyruvate metabolism	2	0.0060371	0.017249
Taurine and hypotaurine metabolism	2	0.015772	0.03943
Alanine aspartate and glutamate metabolism	4	0.020867	0.049098
Arginine and proline metabolism	3	0.026161	0.038135

## Data Availability

Data are contained within the article and Supplementary Materials.

## Supplementary Materials

The following supporting information can be downloaded at: https://www.mdpi.com/article/10.3390/antiox14060753/s1, Figure S1: Quantitative analysis CoQ10; Table S1: Cross-validation performed with 10-fold methods reported the accuracy, R2 and Q2 value of three components; Table S2: Enrichment metabolomics results performed using MetaboAnalyst representing the number of metabolites for each pathway, Raw p and the adjustment according to Holm Bonferroni test and False Discovery Rate values (FDR). Only pathways reporting hits >1 and *p*-value < 0.05 were considered significant.
