# Supplementary material for: Impact of a Formulation Containing Chaga Extract, Coenzyme Q10, and Alpha-Lipoic Acid on Mitochondrial Dysfunction and Oxidative Stress: NMR Metabolomic Insights into Cellular Energy
Source: Antioxidants (Basel). 2025 Jun 18;14(6):753. doi: 10.3390/antiox14060753 (PMC12189963; doi:10.3390/antiox14060753)
Supplement: Supplementary file 1 [file antioxidants-14-00753-s001.zip › antioxidants-3674342-supplementary.pdf]

Impact of a Formulation Containing Chaga Extract, Coenzyme Q10, and Alpha-Lipoic Acid on Mitochondrial Dysfunction and Oxidative Stress: NMR Metabolomic Insights into Cellular Energy

Maria D'Elia et al.

Supplementary material

Quantitative analysis CoQ10

Coenzyme Q10 (CoQ10) was determined by UHPLC-UV analysis. The analysis was performed on a Platin Blue UHPLC system (KNAUER GmbH, Berlin, Germany) equipped with a diode array detector. The chromatographic separation was performed on a Kinetex C18 column (100 x 2.1 mm I.D., 2.6 µm; Phenomex, Bologna, Italy) was used a flow rate of 400 µL min-1 at 30 °C. Volume of the injection was 5 µL. The mobile phase consisted of MeCN:THF:Milli-Q water (50:40:5, v/v/v) was utilized in isocratic elution mode. The UV chromatograms were recorded at 265 nm and calibration external standard method was used to quantify CoQ10 in sample. Five different Coq10 concentrations were prepared diluting with mobile phase appropriate volumes of CoQ10 stock solution (2 mg mL^{-1}). Linearity of calibration curves were evaluated in the concentration range of 50-500 µg mL^{-1} and triplicate injections for each level. UV peal areas of the external standards were plotted against the corresponding standard concentrations (µg mL^{-1}). The resulting regression curves were analyzed using analysis of variance (ANOVA), and a linear model was found to be appropriate over the tested concentration range (y= 4695,2x + 241295; R^2= 0.9984). The sample was diluted with mobile phase for quantification analyses. The amount of CoQ10 was finally expressed as milligrams per gram of the formulation, as the mean of triplicate determinations.

Figure S1. UHPLC-UV profiles of CoQ10 in sample (265.1 ± 10.9 mg g^{-1}) (thin) and reference standard (250 µg mL^{-1}) (dashed) at 265 nm.

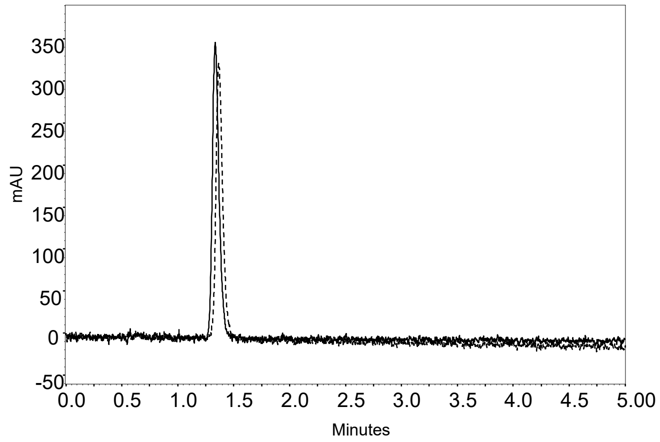

Table S1. Cross-validation performed with 10-fold methods reported the accuracy, R2 and Q2 value of three components.

Endometabolome	PC1	PC2	PC3
ACCURANCY	1.0	1.0	0.98
R2	0.89	0.87	0.76
Q2	0.91	0.88	0.67
**Esometabolome**	**PC1**	**PC2**	**PC3**

**ACCURANCY**	1.0	1.0	1.0
**R2**	0.92	0.94	0.80
**Q2**	0.78	0.85	0.65

Table S2. Enrichment metabolomics results performed using MetaboAnalyst representing the number of metabolites for each pathways, Raw p and the adjustment according to Holm Bonferroni test and False Discovery Rate values (FDR). Only pathways reporting hits >1 and p.value<0.05 were considered significant

	**Hits**	**Raw p**	**Holm p**	**FDR**
Pantothenate and CoA biosynthesis	4	9,56E-05	3,82E-03	3,82E-03
Glyoxylate and dicarboxylate metabolism	6	4,60E-02	0.00017945	9,20E-01
Ubiquinone and other terpenoid-quinone biosynthesis	1	4,63E-01	0.0017591	0.00046293
Tyrosine metabolism	3	4,63E-01	0.0017591	0.00046293
Primary bile acid biosynthesis	1	0.00052912	0.019048	0.0035275
Lipoic acid metabolism	3	0.00052912	0.019048	0.0035275
Porphyrin metabolism	2	0.0014638	0.04977	0.0079534
Histidine metabolism	3	0.0015907	0.052493	0.0079534
Glutathione metabolism	5	0.0018923	0.060553	0.0084101
Fatty acid degradation	1	0.0025678	0.079602	0.0096374
Phenylalanine metabolism	2	0.0028912	0.086736	0.0096374
Phenylalanine tyrosine and tryptophan biosynthesis	4	0.0028912	0.086736	0.0096374
Glycolysis / Gluconeogenesis	1	0.0060371	0.16904	0.017249
Pyruvate metabolism	1	0.0060371	0.16904	0.017249
Taurine and hypotaurine metabolism	1	0.015772	0.41007	0.03943
Thiamine metabolism	1	0.015772	0.41007	0.03943
Arginine and proline metabolism	3	0.026161	0.6017	0.058135
Purine metabolism	4	0.040332	0.84698	0.080665
Nitrogen metabolism	2	0.048442	0.92039	0.088076
Butanoate metabolism	2	0.056621	1	0.098472
Cysteine and methionine metabolism	4	0.089624	1	0.14937
Arginine biosynthesis	4	0.15954	1	0.24493
beta-Alanine metabolism	2	0.16533	1	0.24493
D-Amino acid metabolism	1	0.28616	1	0.3947
Sphingolipid metabolism	1	0.28616	1	0.3947
Fructose and mannose metabolism	1	0.42485	1	0.5332
Amino sugar and nucleotide sugar metabolism	1	0.42485	1	0.5332
Biotin metabolism	1	0.44606	1	0.5332
Propanoate metabolism	1	0.45918	1	0.5332
Nicotinate and nicotinamide metabolism	1	0.47988	1	0.5332

Galactose metabolism	2	0.47988	1	0.5332
Starch and sucrose metabolism	2	0.47988	1	0.5332
Tryptophan metabolism	1	0.58196	1	0.60655
Pyrimidine metabolism	1	0.59139	1	0.60655
Lysine degradation	2	0.70663	1	0.70663
